# Phenotypic heterogeneity in a batch culture of *Chlamydomonas reinhardtii* with different light tolerances

**DOI:** 10.1371/journal.pone.0330144

**Published:** 2026-05-14

**Authors:** Gaganpreet K. Gill, Dion G. Durnford

**Affiliations:** Department of Biology, University of New Brunswick, Fredericton, New Brunswick, Canada; University of Innsbruck, AUSTRIA

## Abstract

Genetic diversity of populations is essential for generating phenotypic variation to allow a flexible response to a shift in environmental conditions. Compared to genetically diverse populations, you would predict that cultures of genetically identical individuals grown in the lab would have low phenotypic heterogeneity. However, we isolated two subpopulations of genetically identical individuals from an exponentially growing batch culture of the microalga *Chlamydomonas reinhardtii* using Percoll step-gradients. The culture fractionated into a low-density, Top fraction and a high-density, Bottom fraction. These subpopulations displayed several phenotypic differences, including size, protein content, the amount of chlorophyll per cell, and photosynthetic performance. Because of the variation in pigment content and photosynthetic performance, we tested the hypothesis that there are differences in their tolerance to light stress. Following high-light stress, the Bottom subpopulation was more resistant to photodamage, had a greater capacity for light dissipation, and had a minimal photoacclimation response to high light, compared to the Top subpopulation. The Bottom population also had a greater resistance to exogenously induced singlet oxygen stress mediated by rose bengal. We hypothesize that these subpopulations are derived from stochastic mechanism where the Bottom subpopulation has activated a general high-light stress response pathway as part of a “bet-hedging” strategy that could give it a fitness advantage with a shift towards a light-stress environment.

## Introduction

Genetic and phenotypic diversity are crucial for microbial populations to acclimate to their environments and adapt to new ecological niches. Natural populations coexist within communities that are shaped by biotic interactions between different species and abiotic factors, such as chemical or physical properties of the environment that change seasonally or unpredictably [1,2]. Environmental heterogeneity is expected to drive genetic and species diversity in populations [1,3]. Unique niches in these heterogeneous environments induce selective pressures, whereby advantageous traits may become more prevalent, driving survival, adaptation, and evolution of phenotypically diverse populations living in naturally dynamic environments [1].

Unexpectedly, phenotypic variation can occur within isogenic populations that are grown under the same environmental conditions. This phenotypic heterogeneity has been observed in a variety of microorganisms. This heterogeneity could come from a variety of sources. Differences in the abiotic environment within a population would clearly produce phenotypic variation. Phenotypic variation can also stem from being in different stages of the cell cycle [4] or the result of asymmetric separation of cell components during cell division that can produce phenotypic differences. In *Escherichia coli* (*E. coli*), the asymmetric distribution of pole proteins during division can give rise to physiologically younger cells with newly synthesized poles or older cells with the original pole composition [5]. The “older” cells have a slower growth rate and reduced resilience compared to the “younger” cells. Finally, phenotypic variation in individuals can come about via molecular noise, that intrinsic stochastic variation in cells. Here, small variations in regulatory molecules can have effects on the phenotype [6], or where epigenetic regulation of regulatory proteins created phenotypic variation, as observed with yeast adhesion proteins [7]. A number of single-cell transcriptome studies in *Chlamydomonas* have demonstrated detectable variation in gene expression in batch cultures [8,9].

Phenotypic heterogeneity is also observed in yeast where oxidatively damaged proteins are unevenly distributed between mother and daughter cells during binary fission in synchronized cells [10,11]. The unequal distribution of damaged proteins ultimately “sacrifices” one daughter cell so the other can thrive [12–15]. Daughter cells inheriting damaged proteins age more rapidly, have longer generation times, and have reduced longevity as compared to their “younger” siblings [10,11]. In yeast, asymmetrical distribution can also include the concentrations of metabolites, signaling pathway components [16–19], and damaged cellular components, such as peroxisomes [20], that can lead to cell-to-cell heterogeneity in isogenic populations. This has the effect of creating individuals in a population with different functional capacities, and a heterogeneous population from genetically identical individuals.

The nature to which this functional heterogeneity exists in other model eukaryotic systems, such as microalgae, is unclear, especially with respect to the functional significance. For instance, with the microalga *Chlamydomonas reinhardtii* (*C. reinhardtii*), isogenic batch cultures are commonly used in experiments, and despite these cultures starting from a single individual, *Chlamydomonas* cultures have variation in traits such as lipid content, cell size, starch metabolism, gene expression, and growth rates [8,21,22]. Lee et al. [21] observed stochastic heterogeneity in lipid content by encapsulating single *Chlamydomonas* cells in alginate hydrogel microcapsules and stained them with the lipid-binding fluorescence stain, BODIPY, revealing significant variation. Similarly, Garz et al [8] used second harmonic generation signals and laser scanning microscopy to quantify starch content per cell in synchronized, isogenic cultures and found that metabolic processes related to starch synthesis and degradation are major contributors to the heterogeneity. These findings suggest that intrinsic factors can drive phenotypic diversity in isogenic cultures of *Chlamydomonas*.

Heterogeneity in *Chlamydomonas* has also been observed as variations in growth dynamics. Despite using isogenic *Chlamydomonas* batch cultures in consistent conditions, Damodaran et al. [22] found significant growth-rate heterogeneity using a millifluidic drop analyzer (a device that tracks the number of cells over time) to assess the growth kinetics. Using this device, the authors identified two subpopulations – the major subpopulation was termed as, “fast-growing” cells, and the minor subpopulation as “slow-growing” with doubling times of 7–11 hours and 12–17 hours, respectively. Damodaran et al. [22] suggests this may be due to some isogenic cells expressing an active dividing state, while others express a restricted dividing state. These studies collectively show that *Chlamydomonas* populations, like bacterial and yeast cultures, develop heterogeneity and phenotypic diversity due to unknown, intrinsic mechanisms.

Building on the understanding of phenotypic heterogeneity in microorganisms, we wanted to investigate this phenomenon in an isogenic batch culture of *C. reinhardtii* cells with the goal of isolating different subpopulations, determine how they phenotypically differ, and determine whether the phenotypic variation was functionally significant. We attempted to physically isolate *Chlamydomonas* subpopulations using Percoll density gradient centrifugation, a technique used to separate microalgal cells based on buoyant density [23,24]. Here we describe the separation of two populations that have differences in macromolecular composition, growth, photosynthesis and respiration. Importantly, these subpopulations showed differences in their tolerance to high light, suggesting they may have a different fitness under changing environmental conditions.

## Materials and methods

### Growing and synchronizing batch cultures

*C. reinhardtii* strain CC125 was obtained from the *Chlamydomonas* Resource Center at the University of Minnesota (https://www.chlamycollection.org/). Cultures were maintained on Tris-Acetate Phosphate (TAP [25]) agar plates under continuous light at room temperature until ready for use.

To ensure an isogenic isolate, we streaked the culture on a TAP plate and isolated a single colony for further propagation. Liquid starting cultures were initiated by aseptically inoculating cells from the TAP agar plate into a 125 ml Erlenmeyer flask containing 35 ml of sterile TAP liquid media. This starting culture was closed with a foam stopper and wrapped with aluminum foil and grown for four days with constant shaking in a 13-hour light and 11-hour dark (13/11-hour L/D) cycle [26]. The light intensity of the growth chamber was 75 µmol quanta m^-2^s^-1^ using cool-white, fluorescent bulbs, and the cultures were grown on a shaker at 24°C inside a growth chamber. The starter culture was then used to inoculate new cultures in fresh TAP media at a starting cell abundance of 3 x 10^5^ cells ml^-1^. These experimental cultures were returned to the same growing conditions for two days. Unless otherwise indicated, experimental cultures were harvested four hours into the light period (L4) two days following culture dilution.

### Percoll density gradient centrifugation

Density gradient centrifugation using a Percoll step gradient (Sigma-Aldrich, Saint Louis, U.S.A.) was used to separate subpopulations of cells from cultures. The gradients were made by overlaying 2 ml of 40% Percoll™ (Cytiva, U.K.), 6.5 ml of 25% Percoll, and 3 ml of 5% Percoll in a 13.2 ml open-top, ultra-clear ultracentrifuge tube (Beckman Coulter, Inc, Fullerton, CA, U.S.A.). Percoll solutions were diluted with water. Gradients were loaded with a total of 2.5 x 10^7^ cells in a 0.5 ml volume and centrifuged at 7885 x g for 30 minutes at 24°C using a SW41 rotor in an Optima XE-90 Ultracentrifuge (Beckman Coulter, Inc, Fullerton, CA, U.S.A.). Subpopulations (or “bands”) were collected using a syringe and needle and kept in sterile falcon tubes.

The subpopulations were washed with media from the original culture (spent media) to remove any residual Percoll and cells collected by centrifugation at 8050 x g for 15 minutes (15°C). Pelleted cells were then resuspended in known volumes of spent media and used for different measurements.

### Cell abundance, Chlorophyll, and starch quantification

A sample of cells were fixed with Lugol’s iodine [27] and counted using a Neubauer hemocytometer under a light microscope.

Chlorophyll was extracted from 2 x 10^6^ cells in 1 ml of 100% methanol and pigments quantified by measuring absorbances at 470, 652, 665, and 750 nm using a Beckman Coulter DU720 UV-Vis spectrophotometer as previously described [28].

For starch analysis, the starch pellets were thawed, suspended in 200 µL of 80% ethanol, and boiled for 20 minutes to solubilize the pellet. Starch content was determined using a Total Starch Assay Kit (AA/AMG Megazyme, Ireland) according to the manufacturer’s instructions and as described [28].

### Protein extraction and quantification

Samples of each subpopulation were collected by centrifugation (15,000 x g for 10 minutes) and the pellets containing whole-cells were stored at −80°C until ready for analysis. Protein extraction and quantification have been previously described [29].

### Confocal fluorescence microscopy

A qualitative assessment of lipid droplets in the subpopulations was done using nile red (Sigma) and confocal microscopy. Samples of each subpopulation (1 x 10^6^ cells ml^-1^) were fixed with 10 µL of 4% paraformaldehyde (PFA) and stained with nile red (Sigma) to a final concentration of 1 µg/ml (in methanol) and incubated in the dark for one hour. The images were captured using a Leica TCS SP8 confocal LSM (analyzed using LAS-X software) and processed using ImageJ (Fiji). Lipid droplets were visualized using an excitation laser 552 nm, and emission at 500–610 nm. Chlorophyll autofluorescence was simultaneously visualized using an excitation laser 488 nm, and emission at 650–710 nm.

### Chlorophyll fluorescence and Photosynthesis Measurements

Photosynthetic efficiency was evaluated by measuring chlorophyll fluorescence using a pulse amplitude modulated PAM101 fluorometer (Walz, Germany). Each subpopulation was acclimated in the dark on a shaker for 15 minutes (room temperature), then a minimum of 2 x 10^6^ cells were filtered onto 25 mm glass microfiber filters (GF/C^TM^, Whatman ^TM^, GE healthcare life sciences, U.K.). Cells were subjected to a saturating light pulse (0.8 s) of 1900 μmol photons m^-2^ s^-^^1^ to measure the maximum quantum efficiency of PSII (Fv/Fm), which can indicate whether the cells were in a stressed state.

Induction curves were also conducted as described [30], to assess non-photochemical quenching (NPQ). After using the saturating pulse to measure Fv/Fm, the filtered cells were exposed to an actinic light of 830 μmol photons m^-2^ s^-1^ while applying a saturating pulse every minute for 13 minutes. The saturating pulses were 0.8 s of 1900 μmol photons m^-2^ s^-1^ light. After 13 pulses, the actinic light was turned off, and the recovery period proceeded for 20 minutes where six saturating pulses were applied at increasing time intervals. Non-photochemical quenching was calculated using the classic equation ((Fm-Fm’)/ Fm’).

Photosynthetic activity was evaluated by measuring oxygen evolution rates using an oxygen electrode with the Oxygraph system (Hansatech Instruments Ltd, U.K.). Each sample was first acclimated under a low-light intensity (68 μmol quanta m^-2^s^-1^) for 5 minutes at 25°C, then the oxygen evolution of cells was measured in complete darkness for 1 minute at 25°C (which estimates the amount of oxygen consumption through respiration). Samples were then exposed to increasing light intensities every minute, until a maximum of 1220 μmol quanta m^-2^s^-1^. The oxygen evolution rates were calculated using O2View software (Hansatech Instruments Ltd) and the data was normalized to cell number, then plotted as a Photosynthesis-Irradiance (PI) curve.

### Flow Cytometry: Cell size

A S3e Cell Sorter (Bio-Rad Canada) was used to measure the cell sizes of subpopulations. Forward scatter (FSC) and side scatter (SSC) measurements were taken of a minimum 1 x 10^5^ cells of each subpopulation. The average cell size of the subpopulations was estimated using a standard calibrated microsphere bead set (Spherotech Inc., USA) as previously described [28]. Using FlowJo^TM^ Software, an initial gate was created by selecting the population of interest on the linear FSC-Area (A) and SSC-A density plot (S1 Fig, panel A). From the gated population, doublets were excluded by further gating the dense population of events along either a linear FSC-Height (H) and FSC-W density plot, or a linear FSC-A and FSC-H density plot (S1 Fig, panel B). The average modes for unique FSC-W scale values were used to generate a standard curve (S2 Fig), and the subpopulation’s cell diameters were calculated using the linear regression line.

### Flow cytometry: DNA content

DNA content was analyzed by staining cells with a double-stranded DNA-binding fluorescent stain, Vybrant^TM^ DyeCycle^TM^ Green stain (5 mM in DMSO) [31]. This stain is excited by a 488 nm laser with a 534/34 band pass filter. Unstained samples were used as a control to account for background fluorescence.

### Growth rate

Relative growth rates over the first two days of transfer to fresh media were determined using absorbance at 750 nm (A750) in cultures grown in 12-well microwell plates. Subpopulations were diluted with sterile fresh media to a final concentration of 3 x 10^5^ cells ml^-1^ and two ml aliquots were placed into individual wells of the microwell plates. Samples were grown for three days (13/11-hour L/D cycles, 24°C) on a shaker and the absorbance was measured at 750 nm using a SpectraMax® M3 Multi-Mode microplate reader (Molecular Devices, U.S.A.) at least twice a day as a proxy for cell abundance. Growth rates were calculated using the equation:


$$\begin{array}{c} Growth\;Rate\;(\mu )=\frac{ln(\text{N}\text{2})-ln(\text{N}\text{1})}{dT} \end{array}$$

For a qualitative assessment of growth yield, the subpopulations were diluted to a starting concentration of 4 x 10^6^ cells ml^-1^ and 10 µl of each sample was pipetted onto a solid TAP media plate and grown for four days to look for visual cues for differences in their growth.

### Rose Bengal treatment

Rose Bengal (RB) is a photosensitizer that induces singlet oxygen formation when exposed to light [32]. Samples of each subpopulation (200 µl of 1 x 10^6^ cells ml^-1^ suspensions) were added to 96-well microplates with 40 µl of increasing concentrations of RB. The plates were incubated either in the dark (control) or under light (75 µmol quanta m^-2^s^-1^) for 1.5 hours, then cells were collected and centrifuged for 10 minutes at 15,000 x g (13°C). The supernatants containing RB were removed and the pelleted cells were resuspended in 240 µl of fresh TAP media. For a qualitative assessment, 5 µl of the samples were pipetted onto a solid TAP media plate and grown for up to 4 days under continuous light (56 µmol quanta m^-2^s^-1^).

### Statistical analyses

Data was imported into Microsoft Excel^TM^ for graphing and computing Student *t*-tests (two-tailed, two-sample equal variance). Two-way ANOVAs and Tukey’s HSD tests were performed using RStudio [33].

## Results

### Two subpopulations separated based on buoyant density

We explored heterogeneity of isogenic *Chlamydomonas* batch cultures by fractionating cells based on buoyant density. The cultures were harvested at Day 2, four hours into the light period (L4). These cells are growing exponentially, thus avoiding any significant age-related differences in the culture (S3 Fig).

**Fig 1:**
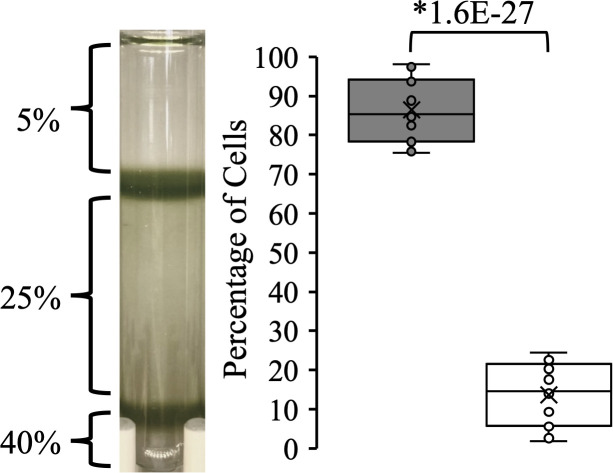
Separation of a batch culture into two subpopulations using a Percoll step-gradient. Proportions of the Top (gray box) and Bottom (white box) subpopulations are shown as box and whisker plots with the median shown as the center line. Individual data points (circles, n = 19) are shown. Asterisks indicate significant differences between the bands with the *p*-value indicated (Student’s *t*-test).

When we centrifuged the culture on a Percoll step gradient (5-25-40%) it consistently fractionated into two subpopulations: a lower density population (Top) at the interface of the 5–25% Percoll boundary; and a higher density population (Bottom) at the 40%-Percoll step. The Top subpopulation was significantly more abundant with 86% ± 7.9% SD of the cells compared to the Bottom subpopulation (with 14% of the cells; *p* = 1.6 x 10^-27^, Student’s *t*-test) (Fig 1).

**Fig 2:**
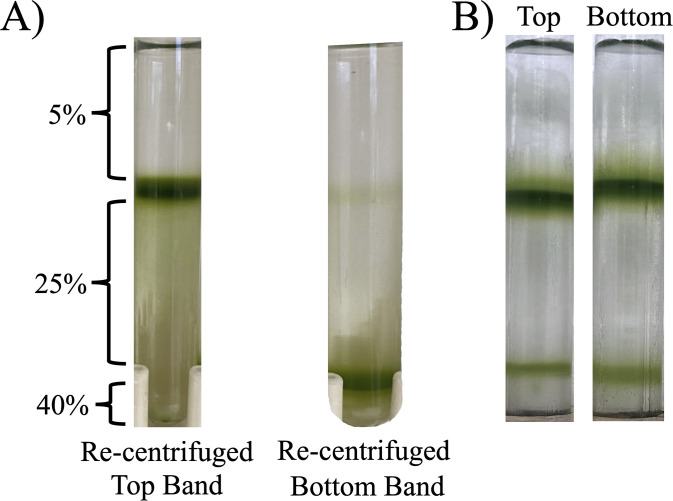
Stability of the subpopulations. A) Each subpopulation collected and re-centrifuged onto new Percoll step-gradients, and B) Top and Bottom subpopulations were collected and inoculated into fresh TAP media liquid cultures (35 ml) starting at 5.4 x 10^4^ cells ml^-1^. Subpopulations were grown for three days and loaded onto new Percoll step-gradients.

To determine the stability of the fractions, the bands were collected, washed, and re-centrifuged onto new Percoll step-gradients (Fig 2A). In this test, cells from the Top subpopulation again fractionated at the 5–25% Percoll interface and the Bottom subpopulation at the 25–40% interface, except for a trace amount in the 5–25% interface. This confirmed a robust separation based on density and helped confirm no additional subpopulations diverged from either band [34,35].

Although we started this study by isolating a single colony to ensure the CC125 culture was isogenic, we tested the possibility that the Top and Bottom subpopulations may be fixed phenotypes in the culture, whether due to genetic variation or epigenetic modification [2,36]. Each subpopulation was collected and inoculated into new flasks with TAP media and grown for three days under the same 13/11-hour L/D regime. In each case, a Top and Bottom fraction were identified in the same, approximate relative distribution of both subcultures (Fig 2B).

**Fig 3:**
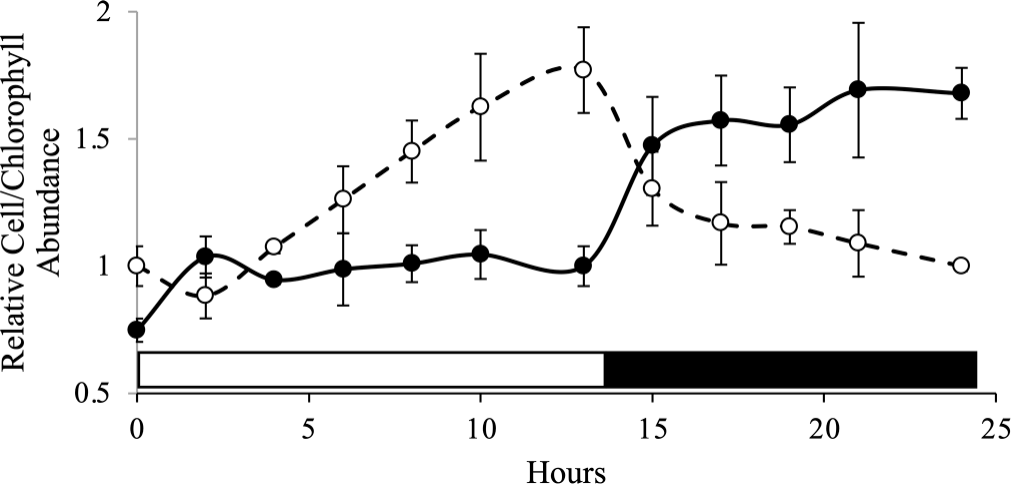
Relative cell abundance and chlorophyll content per cell over one L/D cycle in a Day 2 batch culture. Relative number of CC125 cells per ml (closed circles, solid line; n = 3,± SD for all except 13 hours (n = 6) and 24 hours (n = 2)). Relative amount of total chlorophyll content (*a + b*, pg cell^-1^) (open circles, broken line). The open bar on the bottom of the graph indicates the light period, and the closed bar indicates the dark.

**Fig 4:**
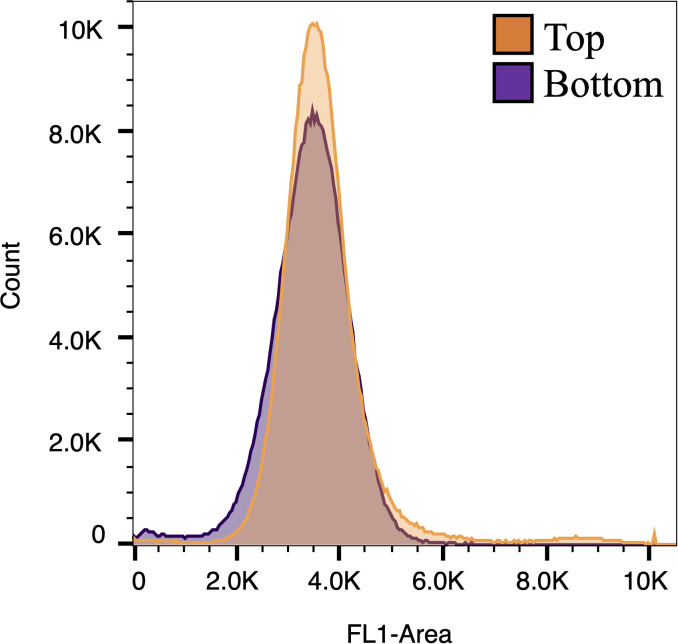
DNA fluorescence of the Top and Bottom subpopulations using flow cytometry that were harvested at L4 on Day 2. Subpopulations were stained with Vybrant DyeCycle Green and the histograms of DNA Fluorescence-Area (FL1-Area) for both subpopulations are overlayed.

To test if the subpopulations represented different stages of the cell cycle we first monitored the Day 2 culture over a 24 hr period of the L/D cycle to see if the culture was synchronized. Other than an increase from the start of the light period to hour 2, there was no significant increase in cell abundance during the light period (Fig 3). At the start of the dark period, however, cell abundance increased about 1.5-fold indicating about half of the cells divided. The cells did accumulate chlorophyll over the light period, suggesting cells were getting larger, and the chlorophyll per cell declined when cells started to divide in the dark phase (Fig 3). Overall, this indicated that the culture was reasonably synchronized. To specifically test whether the Top and bottom fractions differed in DNA content, we stained each subpopulation with Vybrant^TM^ DyeCycle^TM^ Green stain to assess DNA content per cell using flow cytometry. We did not observe a difference in DNA content between the Top and Bottom fractions that would suggest variation in stage of the cell cycle or explain differences in buoyant density (Fig 4).

**Fig 5:**
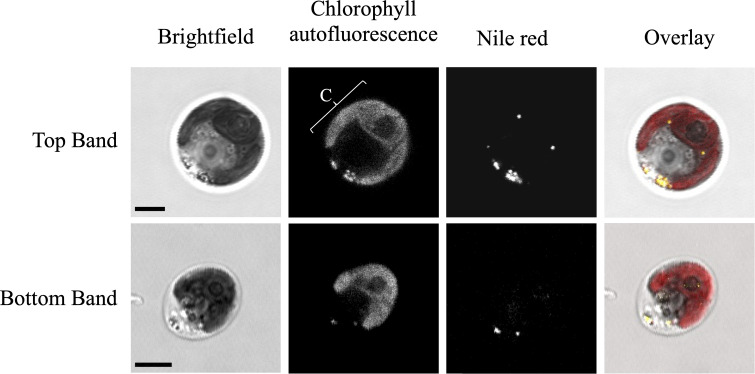
Morphology of the most observed cell types in each subpopulation. Chlorophyll autofluorescence microscopy shows the chloroplast (C) and shape of the plastid, and nile red-staining of each subpopulation detects lipid droplets using fluorescence microscopy. For the overlay images, nile red (yellow) and chlorophyll autofluorescence (red) are shown. Scale bars = 5 μm. Images were enhanced for brightness and contrast using ImageJ.

To decipher the differences between the Top and Bottom subpopulations, we initially focused on morphological characteristics, such as cell size and shape. Using brightfield microscopy, we surveyed 300 cells from each fraction and observed 89% of the cells in the Bottom fraction were predominantly ellipsoidal, compared to 68% in the Top fraction. While there was clearly a variation in morphology in each subpopulation, the cells in Fig 5 were chosen from a dozen confocal microscopy images and represent the cell size and shape most observed. The chlorophyll autofluorescence panels show the typical arrangement of a cup-shaped chloroplast along the bottom of the cells, and the ellipsoidal-cells had smaller plastid lobes. In Fig 4, nile red was used to stain lipid droplets in the cells and its fluorescence was examined using confocal microscopy. The rounder cells in the Top fraction had more lipid droplets compared to the more elliptical cells in the Bottom fraction.

The different populations had clear differences in morphological and biochemical properties. Using flow cytometry, we measured the cell diameter (Table 1), and on average the Top subpopulation was significantly larger than the Bottom population. Assuming the volume of a sphere, this equates to volume difference of 14% between the two populations. The total protein content per cell was about 40% greater in the Top subpopulation than the Bottom (Table 1). The total chlorophyll content per cell was similarly higher in the Top subpopulation on a per cell basis, though the ratio of Chl *a* and *b* in the two subpopulations was identical (Table 1). Despite the differences in protein and chlorophyll content, there were no differences in the amount of starch per cell between the two subpopulations (Table 1).

**Table 1:** Size and biochemical characteristics of the Top and Bottom subpopulations.

Characteristic	Top	Bottom
Cell diameter (µm)	9.1 ± 0.4 *	8.7 ± 0.3
Total chlorophyll *a + b* (pg cell^-1^)	6.0 ± 1.1 *	4.0 ± 1.3
Chl *a* (pg cell^-1^)	4.4 ± 0.8 *	2.9 ± 0.9
Chl *b* (pg cell^-1^)	1.6 ± 0.4 *	1.0 ± 0.3
Chl *a/b* ratio	2.9 ± 0.3	2.9 ± 0.3
Total protein (pg cell^-1^)	171 ± 15.6 *	110.1 ± 19.7
Total starch (pg cell^-1^)	2.5 ± 1.3	1.6 ± 1.0

* significantly different from Bottom subpopulation (*p* < 0.006, Student’s *t*-test). Error bars are ± SD (n = 12 for cell diameter; n = 19 for chlorophyll content; n = 5 for protein and starch content)

## Functional characteristics of the subpopulations

### Growth rate

**Fig 6:**
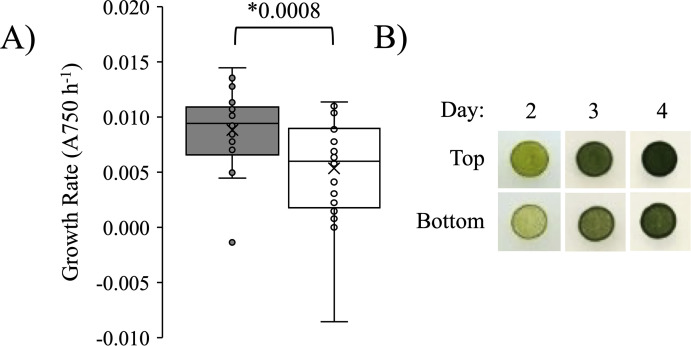
Growth differences between the Top and Bottom subpopulations. A) Average growth rates after growth in liquid media for 38–42 h. The boxes represent 50% of the data and the whiskers show the distribution of the upper and lower 25%. Asterisks indicate significant differences between the bands with the *p*-value indicated (Student’s *t*-test). Individual data points (Top band, filled circles, n = 26) (Bottom band, open circles, n = 25) are shown. B) Subpopulations grown on TAP agar plates for 2–4 days as a qualitative assessment of growth rate.

To determine if there were differences in growth rate between the Top and Bottom subpopulations, we conducted a quantitative growth assay in liquid media and a qualitative assay on agar plates. The Top subpopulation had an 60% greater average growth rate over two days, where the Top population had a significantly higher initial growth rate of 0.0088 A750 hr^-1^ while the Bottom population grew at 0.0053 A750 hr^-1^ (*p* = 0.0008, Student’s *t*-test) (Fig 6A). When equal numbers of cells were plated onto a TAP plate and observed over four days, the growth differences are visible and in agreement with the liquid assay (Fig 6B).

### Photosynthesis

There were also differences in photosynthetic performance between the subpopulations and the Top subpopulation had a significantly greater maximum photosynthetic rate (Pmax) on a per cell basis (Table 2; *p* = 0.001, Student’s *t*-test) and a greater light-use efficiency (α) (*p* = 0.002, Student’s *t*-test), which was determined by the initial slope of the PI curve [37]. The dark oxygen consumption rate, which is a proxy for mitochondrial respiration, was almost double the amount in the Top subpopulation than in the Bottom subpopulation (*p* = 0.05, Student’s *t*-test), suggesting a greater metabolic activity in the Top fraction, potentially explaining the faster growth rate. These differences were largely masked when the parameters were normalized based on total chlorophyll (Table 2).

**Table 2:** Photosynthetic parameters of the Top and Bottom subpopulations.

Parameter	Top	Bottom	Top	Bottom
	per cell (fmol O_2_ cell^-1^hr^-1^)		per chlorophyll (fmol O_2_ nmol chl *a + b*^-1^hr^-1^)	
Dark O_2_ consumption rate	−121.4 ± 50.6*	−66.9 ± 18.5	−20.6 ± 9.5	−18.7 ± 1.8
lc	39.4 ± 19.8	32.1 ± 7.0	39.4 ± 19.8	31.7 ± 7.4
α	3.5 ± 0.6 *	2.1 ± 0.4	0.6 ± 0.1	0.6 ± 0.2
Pmax	392.2 ± 25.6 *	247.6 ± 51.8	65.7 ± 3.9	70.8 ± 13.4

*significantly different from Bottom subpopulation, *p* < 0.05, Student’s *t*-test. lc: light compensation point (µmol quanta m^-2^s^-1^); α: light-use efficiency (fmol O_2_ cell^-1^hr^-1^ OR fmol O_2_ nmol chl *a + b*^-1^hr^-1^ per µmol quanta m^-2^s^-1^); Pmax: maximum photosynthetic rate.

### High-light stress tolerance

**Fig 7:**
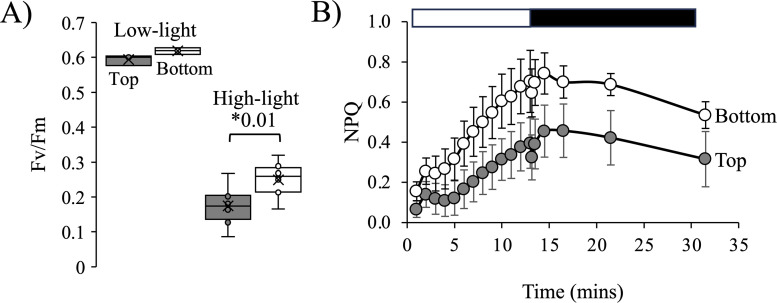
Photosynthetic efficiency and maximum quantum efficiency of PSII in the Top and Bottom subpopulations. A) Subpopulations were exposed to LL (75 µmol quanta m^-2^s^-1^) or HL (530 µmol quanta m^-2^s^-1^) for two hours and all contents of the flasks were measured for their Fv/Fm. The boxes represent 50% of the data and the whiskers the distribution of the upper and lower 25%. Asterisks indicate significant differences between the Top (gray) and Bottom (white) subpopulations with the *p*-value indicated (Student’s *t*-test). B) Average light dissipated by non-photochemical quenching (NPQ) over time for Top (gray circles) and Bottom (white circles) subpopulations (n = 6, ± 2SE). The open bar indicates the hours spent in light, and the closed bar indicates hours spent in the dark.

Considering the differences in photosynthetic rates and chlorophyll levels, we examined the photoprotection and light energy dissipation capacity using chlorophyll fluorescence (Fig 7). We first tested the light tolerance of each subpopulation in a short-term light stress experiment. Cells from each subpopulation were diluted to 2−5 x 10^6^ cells in 10 ml of spent media and placed in 50 ml Erlenmeyer flasks. Subpopulations were incubated in low-light (LL) (75 µmol photons m^-2^s^-1^) or high-light (HL) (530 µmol photons m^-2^s^-1^) for 2 hours with constant shaking, after which the Fv/Fm was determined (Fig 7A). There was no difference in the Fv/Fm between the populations under LL, but there was a small, significant difference in the Fv/Fm after HL-exposure. The Fv/Fm of the Top subpopulation was 0.17 ± 0.05 SD, while the Fv/Fm of the Bottom fraction was 0.25 ± 0.05 SD (*p* = 0.01, Student’s *t*-test). This suggested that the Top subpopulation was more photoinhibited during the HL exposure.

Since there seemed to be differences in tolerance to HL-stress, we assessed the capacity of each population to dissipate light energy by measuring non-photochemical quenching (NPQ) during an equivalent HL-stress. NPQ is a way to bypass light energy away from the core reaction center to protect the photosynthetic apparatus [38]. In these experiments, the Bottom subpopulation showed a significantly greater amount of light energy dissipated through NPQ than the Top subpopulation (Fig 7B). At 10 minutes, the average amount of light energy dissipated through NPQ in the Bottom subpopulation was double (0.605 ± 0.2 SD) that of the Top subpopulation (0.314 ± 0.1 SD, *p* = 0.006, Student’s *t*-test) (Fig 7B). There was also a faster induction of NPQ, throughout the light induction curve for the bottom fraction, implying a greater qE component. During the dark recovery period, both populations showed a similar recovery with evidence for photoinhibition at the end of the recovery period (Fig 7B).

**Fig 8:**
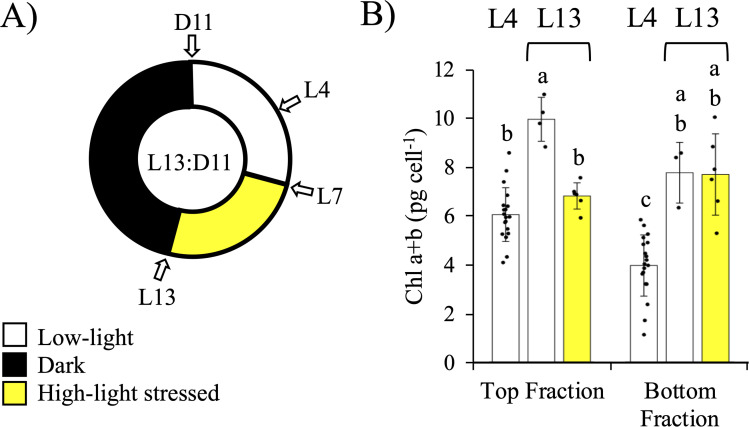
Effects of high-light stress on Top and Bottom subpopulations. A) Diagram illustrating the experimental design with or without HL (531 µmol quanta m^-2^s^-1^) exposure to the batch cultures between L7-L13 on Day 2. At L13, the culture was fractionated on a Percoll gradient to separate out the Top and Bottom fractions. B) Total chlorophyll content (*a* ± *b*) for each subpopulation was measured at photoperiods L4 and L13 (n = 3–6, ± SD) (n = 19 for L4). Individual data points are shown. Bars labelled with different letters indicate significant differences within each panel (*p* < 7e-5 for ANOVA, and *p* < 0.05 for Tukey’s HSD test).

Next, we examined each subpopulation’s capacity to photoacclimate to HL stress over the course of a 13-hour period using chlorophyll content as a marker for photoacclimation state (Fig 8A). In *Chlamydomonas*, there is usually an inverse relationship between chlorophyll content and light intensity they were grown under that can roughly approximate the “acclimated state” of the cells. However, this can be complicated as *Chlamydomonas* cells increase in size over the course of the day [26,39]. As the cell gets bigger, the amount of chlorophyll content per cell also increases (as seen in Fig 3). However, we can follow this increase in chlorophyll content during the light period under different light intensities to assess photoacclimation capacity.

With a light shock experiment (Fig 8A), the Top and Bottom populations responded differently to excess light. Under four hours of LL (L4), the Top fraction had a greater chlorophyll content per cell than the bottom (Fig 8B), as previously noted (Table 1). Continuing under LL conditions, both fractions increased their chlorophyll content over the course of the light period by 69 and 96% for the Top and Bottom subpopulations, respectively. Although, the Top fraction still had the higher chlorophyll content per cell at the end of the day (L13; 22% greater, Fig 8B). When a HL shock was administered starting seven hours into the light period (L7) and lasting until the end of the day (L13), chlorophyll accumulation in the Top population was significantly repressed (6.8 pg cell^-1^ ± 0.5 SD; *p* < 0.05, Tukey’s HSD test) (Fig 8B), while the HL-exposed Bottom fraction’s chlorophyll content remained unchanged compared to its LL control (Fig 8B). There was no significant difference in the total number of cells present for either subpopulation under LL or HL (S4 Fig).

### Oxidative stress tolerance

**Fig 9:**
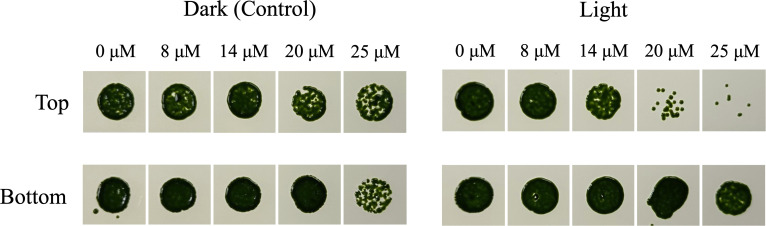
Rose bengal (singlet oxygen) resistance of the Top and Bottom subpopulations. Subpopulations were diluted to a starting concentration of 1 x 10^6^ cells ml^-1^, exposed to rose bengal in the presence or absence (control) of light, and 5 μl of the culture was dispensed onto TAP agar plates. Subpopulations grown on TAP agar plates for 7 days as a qualitative assessment of growth after exposure to singlet oxygen from rose bengal treatment.

We also tested each subpopulation’s ability to tolerate oxidative stress to help decipher differences in the photoprotection pathways seen thus far. We used increasing concentrations of rose bengal (RB), a photosensitizer that induces the formation of singlet oxygen when it is exposed to the light [32,40,41]. In the light, RB ultimately transitions from its ground state (S0) to an excited triplet state (T1) [40]. In the triplet state, RB transfers its energy to ground-state oxygen which excites molecular oxygen into its singlet state (^1^O_2_*). In *Chlamydomonas*, accumulation of singlet oxygen is toxic and can induce photodamage. In this experiment, cells were exposed to increasing concentrations of RB for 1.5 hours in the light or the dark (control), to simulate a progressively stringent singlet oxygen stress. After which, cells were washed and plated onto a fresh TAP plate to screen qualitatively for survival. In the dark, both the Top and Bottom subpopulations were unaffected, as expected (Fig 9). However, in the presence of light the Top subpopulation was considerably more sensitive to the RB-treatment, with obvious effects starting at 14 µM. This indicates that the Bottom subpopulation was less sensitive to singlet oxygen stress.

## Discussion

### Heterogeneity in natural and isogenic microbial populations

Microbial populations in nature will exhibit genetic and phenotypic diversity even when individual cells share the same habitat. But diversity still arises because ecosystems are naturally dynamic, undergoing changes like temperature fluctuations, nutrient depletion, seasonal changes, and shifts in resource availability. To survive, organisms must adapt to these environmental fluctuations which can result in phenotypic diversity amongst cells of the same species. On the other hand, when isogenic cells are grown under controlled laboratory conditions, they are expected to show minimal phenotypic variation because the uniform environment should elicit cells to behave identically across the whole population. However, in our study, we consistently detected at least two subpopulations in batch cultures of *C. reinhardtii* (CC125) using Percoll density gradient centrifugation, which separates cells based on buoyant density.

Previous studies have shown evidence of cellular heterogeneity in isogenic cultures of *Chlamydomonas* through biochemical or physiological characteristics such as cell size, starch content [8], lipid content [21], and growth rates [42]. Similarly, in isogenic bacterial populations, heterogeneity has been identified through asymmetric cell division that causes unequal inheritance of cellular components, such as old cell poles [5,17], or the accumulation of damaged intracellular proteins [15]. The spontaneous formation of persister cells in bacteria is another example of heterogeneity arising from isogenic populations, in which a portion of the population switches to a dormant phenotype that is resistant against antibiotics [43–45]. In isogenic yeast populations, heterogeneity is observed as variations in replicative aging [12], the accumulation of carbonylated proteins [10,11], cell cycle arrest in older cells, and differences in signaling pathways [16,19]. These findings demonstrate how phenotypic variation and cellular heterogeneity are not restricted to natural environments but can also manifest in isogenic populations under controlled conditions, consistent with the results observed in this study.

### Buoyant density differences of Top and Bottom subpopulations are independent of cell cycle stage

It wasn’t clear what was responsible for the differences in buoyant density between the two populations. Buoyant density is a tightly regulated cellular property essential for maintaining key biological processes including division, differentiation, and apoptosis [46]. Cells of the same cell-type typically maintain a consistent density emphasizing its functional importance [47–53]. The observed differences in buoyant density between the Top and Bottom subpopulations may arise from several factors, including variations in water content, osmoregulation [48,54–56], organelle composition [48,57], and metabolic state [46,58–60]. In *Chlamydomonas*, osmoregulation is also mediated by a contractile vacuole (CV) complex that has two CVs in the anterior-end, each of which is formed by the fusion of numerous small vesicles before fluid expulsion [61,62]. Since CV size is linked to cell size in *Chlamydomonas*, with larger cells having longer intervals between systole contractions and a greater pumping rate of fluid expulsion [62,63], the larger Top subpopulation may have higher water content contributing to its lighter buoyant density, but this is something we cannot confirm. Structural differences, such as variations in cell wall or chloroplast composition [57] may also contribute to differences in density between subpopulations.

Additionally, osmolytes are solutes like carbohydrates, polyols, and amino acids, are critical for cellular osmoregulation and stress adaptation [46,58–60]. In *Chlamydomonas*, osmolytes such as glycerol and proline accumulate in response to environmental stresses like HL [64,65] and salinity [66–68]. If the Bottom subpopulation in our study is predisposed of greater levels of osmolytes, like proline, it may contribute to their greater buoyant density and enhanced resistance to HL stress as compared to the Top subpopulation.

While buoyant density can change as a culture ages, as seen in *E. coli* cultures [69], we avoided this complication by consistently harvesting cells on the same day post inoculation and at the same point in the photoperiod (L4). We can’t, however, rule out the chance that cells are in different stages of G1 that may have different densities.

It is unsatisfying not to have a definitive explanation for the basis of the differences in buoyant density between the populations. We did ask whether the lack of cell synchrony could contribute to differences in buoyant density. Synchronization of *Chlamydomonas* is difficult to achieve and even under ideal conditions 29% of the population were in G1 during the division period [70], and the optimal synchronization would be sort-lived as nutrients were depleted. If unsynchronized, you would predict cells in different stages of DNA replication that could contribute to buoyant density differences, as can be observed in some yeast cultures [51]. However, we detected no differences in DNA content between the two subpopulations at the period we isolated cells, which would suggest that the bottom population was not arrested in S or G2 phases. In our experiments, while the cells primarily divided early into the dark period, we estimated only about half of them did so. In fact, that number is likely an overestimate if some cells underwent multiple division cycles, as is common in *Chlamydomonas*. This means that there could be cell size heterogeneity in the culture as a result, even though they are not in the process of actively dividing. However, we would argue that this is unlikely to be the primary reason for the existence of the Top and Bottom populations as we would expect the proportions of the two populations to be more even. It is unlikely that the Bottom fraction represents a small proportion of actively dividing cells since these are slower growing when we isolate them. Also, cell size alone is also not a predictor of buoyant density, which is more related to the composition of the cell. Regardless, the functional differences are real and could offer one population a selective advantage over the other depending on the conditions.

There was also no evidence of genetic differences that would explain the variation in buoyant density, which would be expected to be a more permanent feature of the population. First, we isolated the strain from a single colony to ensure it was isogenic. Second, we looked at the stability of the Top and Bottom phenotype by initiating cultures directly with each fraction. This ultimately led to the similar distribution of the fractions in the population after three days (Fig 2B). This finding aligns with Damodaran et al. [42] who showed inoculating cultures using their fast-growing *Chlamydomonas* subpopulation produced heterogenous progeny, rather than exclusively-fast growing cells. While the reason for the differences in buoyant density are not clear, the evidence points to the idea that cells are consistently responding to culture conditions, intrinsic factors, or some sort of internal signal that is triggering the phenotypic differences [8].

### Functional differences

The different subpopulations have different capacities for light use, which was reflected in the differences in chlorophyll content and photosynthetic parameters. The Top subpopulation has a greater light use efficiency and greater maximal photosynthetic rate. This, coupled with a greater dark respiration, should provide growth advantages especially under light-limiting conditions, and in general, we found a faster yield rate in short-term growth experiments. The Bottom fraction, however, was better able to dissipate excess light, effectively being in a more high-light acclimated state, which agrees with the lower chlorophyll content in the cell, a useful indicator of light acclimation status [71,72].

The Bottom subpopulation had phenotypic characteristics that should give it an adaptive advantage under HL stress. This was confirmed in short-term (2 hour) HL-stress experiments where the Bottom subpopulation showed a greater ability to resist photoinhibition as estimated by looking at the maximum quantum efficiency of PSII after the HL-shock. The Bottom subpopulation also was better able to dissipate excess light energy through non-photochemical quenching (NPQ), a way to de-excite chlorophyll and redirect energy away from the rection center to avoid photodamage [38].

The Bottom subpopulation also showed more resistance in response to high light over a six-hour time frame when applied mid-light phase. At the end of the light period (L13) the Bottom subpopulation exposed to HL continued to accumulate chlorophyll, as did the LL control. But, cells in the Top fraction showed a decline in chlorophyll content per cell, showing a typical response to HL exposure [73–77]. However, the mechanism of this chlorophyll reduction in HL can be complex. While it is likely due to an inhibition of the synthesis and accumulation of chlorophyll-binding proteins in response to HL, it could also be triggered by increasing cell division where the chlorophyll is diluted as the cells divide. This wouldn’t be surprising given that *Chlamydomonas* diverts excess light energy into metabolic pathways that can act as energy sinks [65,78] and could trigger cell division. As more cells reach the critical size [39,79] and undergo cell division by L13. However, we were unable to resolve any significant differences in cell number following HL exposure (S4 Fig). Of course, with the Bottom subpopulation, we didn’t see the decline in chlorophyll, which would imply that compositional and functional differences in the cells, including an enhanced NPQ capacity, minimized light stress such that chlorophyll accumulation was not inhibited over the light period. We also did not observe any differences in cell abundance between the LL and HL samples in the bottom subpopulation, which indicates that cell division was not triggered. Nevertheless, there was a clear difference in the response of the two subpopulations to HL exposure, signifying significant functional differences in their response to light.

In addition to a more muted response to HL stress, the Bottom subpopulation also had an increased tolerance to a singlet oxygen stress induced by rose bengal (Fig 9). When *Chlamydomonas* is exposed to too much light beyond what it can dissipate, there is the inevitable production of ROSs that can damage biomolecules and become toxic to the cell [80]. Cells have a variety of inducible detoxification and repair mechanisms as part of an ROS acclimation response [60,80]. For example, Ledford et al. [32] found enhanced resistance to singlet oxygen in *Chlamydomonas* when cells were exposed to RB under HL, suggesting an overlap between ROS and HL-induced response pathways. The acclimation response is partly regulated by the SAK1 gene in *Chlamydomonas*, which becomes phosphorylated in the presence of singlet oxygen [80,81]. The greater tolerance of RB in the bottom population may mean a more global tolerance of stressors related to excess light, and perhaps the pathways related to ROS detoxification [82], or at least those for singlet oxygen. The enhanced singlet oxygen resistance could also be related to other mechanisms, such as the accumulation of osmolytes or other chemicals that act as ROS scavengers as part of a global HL-stress response mechanism [83–85]. Overall, cells in the Bottom subpopulation have attributes resembling a HL-acclimated state [78,86,87], making them more resistant to HL and singlet oxygen damage.

### Origin of phenotypic heterogeneity

The source of the phenotypic heterogeneity in a batch culture of genetically identical individuals grown under the same conditions is intriguing. Phenotypic variation could arise from cellular aging which contributes to heterogeneity over time [5,17,20] or through the accumulation of damaged molecules [10,11] that mimic aging processes by reducing the fitness and viability of cells over time [88]. While we did use young, two-day old cultures and standardized the harvesting time to make these processes less likely, it’s difficult to rule out such mechanisms. Currently, mechanisms contributing to any cell division asymmetry in *Chlamydomonas*, whether age or stress related, are unknown.

Environmental factors, both abiotic and biotic, can contribute to phenotypic variation in isogenic populations [45,89,90], but in our experiments we attempted to keep conditions in the flask uniform. However, it is difficult to rule out distinct microenvironments in our cultures that could activate specific signal pathways in subsets of the population [2,36,91–94], though we would have predicted more variability between experiments if that were the case [89,90].

Cell-to-cell or neighbor interactions could also drive phenotypic switching. For example, in bacteria autoinducers (signal molecules) can trigger phenotypic-switching response based on the cell abundance. This may, for instance, cause a change in biofilm formation [95–97] or secretion of virulence factors (proteins) [98] and increase the variability in the population. However, such systems are not known in *Chlamydomonas,* to our knowledge.

Finally, stochastic mechanisms, including random gene expression and metabolic fluctuations, play a key role in generating phenotypic heterogeneity [94]. Single-cell transcriptome studies in *Chlamydomonas* have found significant variability in the expression of photosynthesis-related, stress-response, and metabolic genes [8,99]. This variability can be driven by epigenetic regulation or stochastic phenotypic switching [21,99], which allows individual cells to toggle between distinct gene expression states [100], potentially explaining the observed differences in the photoprotective strategies and ROS tolerance between the Top and Bottom subpopulations. For example, the ROS detoxification mechanisms and acclimation responses in the Bottom subpopulation may be linked to stochastic gene regulation of commonly regulated stress-response pathways, that may involve of SAK1 [81], for instance. Further investigation using techniques such as RNAseq and/or single-cell sequencing on the Top and Bottom populations could provide deeper insight into the stochastic mechanisms and underlying phenotypic variation in *Chlamydomonas* batch cultures.

In isogenic cultures of the microalga, *Haematococcus pluvialis*, cells can stochastically switch between mobile and non-mobile phenotypes with different stress tolerances [101]. Random fluctuations in gene expression that present as gene noise can cause phenotypically identical cells to differ in behaviour as well, due to varied protein or enzyme levels, which in turn affects metabolic pathways and other functions [6,102–106]. Although isogenic cells experience a baseline level of genetic noise, being a biological system, they still present as phenotypically identically cells. When this noise surpasses a certain threshold, it can lead to phenotypically distinct subpopulations that are now detectable based on their traits [99,102,107]. These intrinsic stochastic events are important to consider when populations encounter stressful environments, because phenotypic variation increases the likelihood for survival for some individuals based on the phenotype favoured under certain conditions [2,108].

Could the two populations be an artifact of the preparation? It’s possible, and there is precedence for this in the fractionation of red blood cells where cell aggregation affected the banding pattern [35]. However, there is evidence to suggest this is not the case in this study. First, the proportion of the populations after dozens of experiments over two years are remarkably consistent (Fig 1). Second, there are clear biochemical and functional differences that would be difficult to explain simply by an artifact of the preparation (Fig 6-9, Tables 1,2). Third, the fractions are stable, and isolation of each, washing and re-centrifugation leads to the fractionation of the Top and Bottom fraction again (Fig 2), which we predict would change if an artifact of the preparation.

### Evolutionary advantage of phenotypic heterogeneity

If the different subpopulations originate through stochastic changes in regulatory pathways, then there should be a fitness disadvantage as some members of the population are not ideally suited to the environmental conditions, arguably like the Bottom fraction. However, such variation may have a benefit in environments where the conditions change suddenly, thus one subpopulation unfavoured in one environment is favoured in the new environment, a strategy known as bet-hedging [109–111]. Bet-hedging increases a population’s chances of survival, reproductive success, and long-term persistence when environmental conditions are uncertain, fluctuate, or become unfavourable. Without such strategies, isogenic cultures remaining phenotypically identical and respond uniformly to stress could risk extinction if environmental conditions suddenly changed [108].

In bet-hedging, only a portion of the population varies phenotypically, acknowledging the risks and rewards associated with phenotypic diversity. For example, bacterial persistence, where a subpopulation of persister cells that grow slowly can survive lethal doses of antibiotics [43–45]. Even while growing under steady environmental conditions, a small fraction of persister cells are already present before any antibiotics are applied [43]. These persister cells spontaneously switch between normal and slow-growing phenotypes, seemingly to ensure a portion of the population will survive the sudden presence of antibiotics and resume normal growth. This bet-hedging strategy emphasizes how phenotypic diversity helps to spread the risk of the stressor across different phenotypes [109,112], and allowing populations to sacrifice optimal fitness in stable conditions for better resilience against environmental stresses [2,113].

The phenotypic heterogeneity observed in this study may serve as an adaptive bet-hedging strategy, where subpopulations diversify their phenotypic traits to enhance survival under fluctuating environmental conditions, particularly light. In the case of the Top and Bottom subpopulations, there would be a trade-off between growth and stress resistance. Future work should prioritize an analysis on the expression of genes in these subpopulations to identify expression profiles related to a LL or HL-optimized expression, perhaps like the shared expression patterns linked to fitness trade-offs between growth and stress resistance found in yeast [114], supporting the idea that phenotypic diversity arises from stochastic processes that balance competing demands.

## Supporting information

S1 Fig Flow cytometry gating scheme to identify cells of interest. The data shown represents a sample of the Top subpopulation (~2.5 x 10^5^ cells). A) Density plot for the number of events according to FSC-A and SSC-A. This plot was used to create the first gate (or channel) to select for events that represent our cells of interest, “Top band cells.” B) Density plot showing the gated events according to FSC-H and FSC-W. This plot was used to create a secondary gate to exclude doublets. The data from the secondary gate, “single cells,” was used for further statistical analyses.
(TIFF)

S2 Fig Standard curve of the average FSC-W scale values of Spherotech calibration microsphere beads using flow cytometry (n = 2, ± SD). (TIFF)

S3 Fig Growth curve of *C. reinhardtii* batch cultures grown for 10 days. Growth curve describing the average cell abundance (n = 3, ± SD) in *C. reinhardtii* (CC125) cultures grown over 10 days under a 12-hr L/D cycle post-inoculation starting at 3 x 10^5^ cells ml^-1^.
(TIFF)

S4 Fig Relative number of cells in subpopulations after six hours of HL-stress during L7-L13. A) Diagram illustrating HL (531 µmol quanta m^-2^s^-1^) exposure to batch cultures between L7-L13, prior to density gradient centrifugation at L13. B) Relative total number of cells for each subpopulation at L13 (n = 3–5,  ±  SD). There was no significant difference between the LL and HL samples within the same population.
(TIFF)
