# Supplementary figures and images for: Phenotypic heterogeneity in a batch culture of Chlamydomonas reinhardtii with different light tolerances
Source: PLoS One. 2026 May 14;21(5):e0330144. doi: 10.1371/journal.pone.0330144 (PMC13175478; doi:10.1371/journal.pone.0330144)

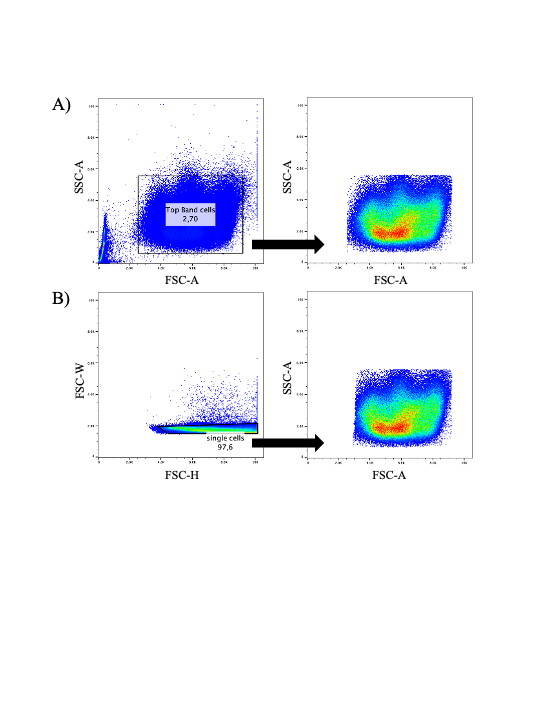

Supplement: S1 Fig — The data shown represents a sample of the Top subpopulation (~2.5 x 10^5^ cells). A) Density plot for the number of events according to FSC-A and SSC-A. This plot was used to create the first gate (or channel) to select for events that represent our cells of interest, “Top band cells.” B) Density plot showing the gated events according to FSC-H and FSC-W. This plot was used to create a secondary gate to exclude doublets. The data from the secondary gate, “single cells,” was used for further statistical analyses. (TIFF) [file pone.0330144.s001.tiff]

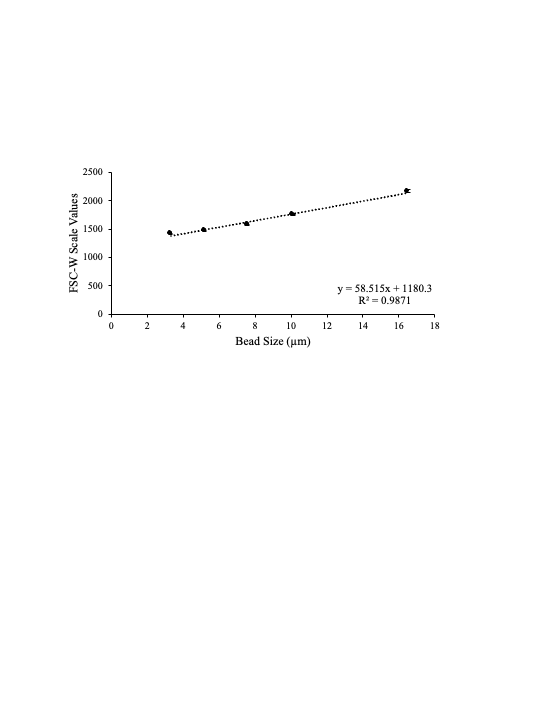

Supplement: S2 Fig — (TIFF) [file pone.0330144.s002.tiff]

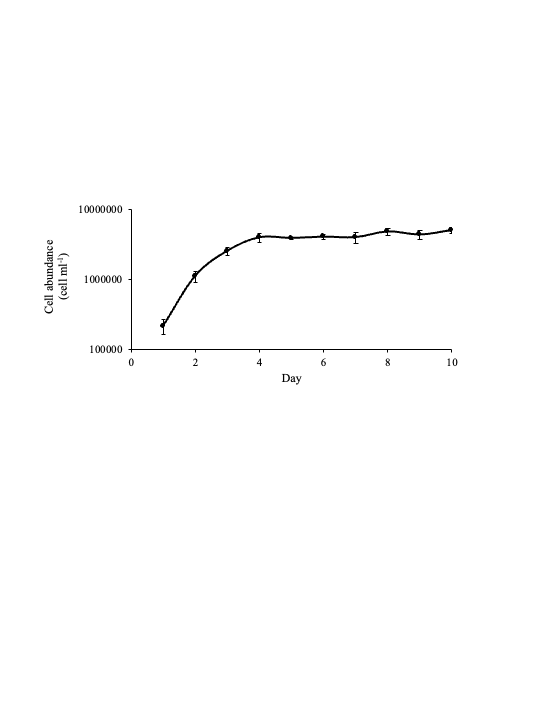

Supplement: S3 Fig — Growth curve describing the average cell abundance (n = 3, ± SD) in C. reinhardtii (CC125) cultures grown over 10 days under a 12-hr L/D cycle post-inoculation starting at 3 x 10^5^ cells ml-1. (TIFF) [file pone.0330144.s003.tiff]

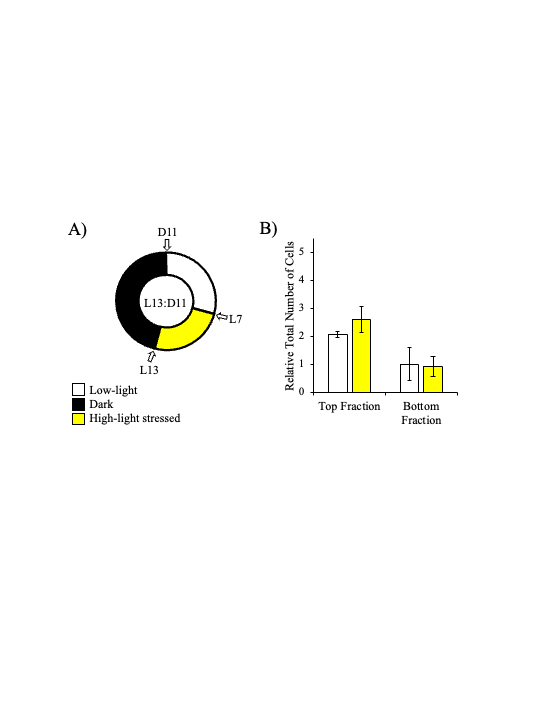

Supplement: S4 Fig — A) Diagram illustrating HL (531 µmol quanta m-2s-1) exposure to batch cultures between L7-L13, prior to density gradient centrifugation at L13. B) Relative total number of cells for each subpopulation at L13 (n = 3–5, ± SD). There was no significant difference between the LL and HL samples within the same population. (TIFF) [file pone.0330144.s004.tiff]
